# Biofilm Producing *Enterococcus* Isolates from Vaginal Microbiota

**DOI:** 10.3390/antibiotics10091082

**Published:** 2021-09-07

**Authors:** Mallika Sengupta, Soma Sarkar, Manideepa SenGupta, Sougata Ghosh, Riya Sarkar, Parthajit Banerjee

**Affiliations:** 1 Department of Microbiology, All India Institute of Medical Sciences (AIIMS) Kalyani, Kalyani 741245, West Bengal, India; 2 Department of Microbiology, NRS Medical College, Kolkata 700014, West Bengal, India; drdssarkar@gmail.com; 3 Department of Microbiology, Medical College, Kolkata 700073, West Bengal, India; manideepa.sengupta2305@gmail.com (M.S.); s_ghosh2006@rediffmail.com (S.G.); 4 Intermediate Reference Laboratory for Tuberculosis, Kolkata 700010, West Bengal, India; riyasarkar26@gmail.com; 5 Department of Microbiology, KPC Medical College, Kolkata 700032, West Bengal, India; banerjeeparthajit52@gmail.com

**Keywords:** vaginal discharge, biofilm, multidrug resistance

## Abstract

Background: *Enterococcus* is an important cause of infection in the hospital as well as in the community. Methods: A prospective study was done in Medical College, Kolkata for a period of 2 years (from January 2018 to December 2019). After obtaining clearance from the Institutional Ethics Committee, *Enterococcus* isolates from cases of vaginitis were included in the study. Identification of *Enterococcus* species was done by Gram stain and conventional biochemical tests along with automated identification by VITEK 2 Compact. These isolates were tested for antimicrobial susceptibility to different antibiotics by Kirby Bauer disc diffusion method and minimum inhibitory concentration (MIC) by VITEK 2 Compact. Interpretation of susceptibility was done according to the Clinical and Laboratory Standards Institute (CLSI) 2017 guidelines. Biofilm detection for *Enterococcus* species was done. Results: During the period of 2 years, 39 isolates of *Enterococcus* spp. were obtained from vaginitis cases. Among these, 27 were *Enterococcus faecalis* and 12 *Enterococcus faecium*. All isolates were highly susceptible to vancomycin, teicoplanin, and linezolid. Biofilm was detected in eight isolates of which five were strong biofilm producer and three moderate biofilm producers. Conclusion: Biofilm production is an important virulence factor in *Enterococcus* isolates from vaginitis.

## 1. Introduction

Vaginitis includes a spectrum of conditions that cause vaginal and, infrequently, vulvar symptoms, like itching, burning sensation, irritation, odor, and vaginal discharge [1]. The vaginal symptoms are often overlooked by women in India, but gradually they are becoming conscious of these symptoms and seek medical advice. The commensal vaginal flora is extremely essential for maintaining proper pH and preventing the colonization of the vagina by other infections and pathogens. Certain commensal flora like lactobacilli play a major role as the first line of defense of the body. When the initial barrier is broken down, the vagina might be colonized by pathogens which might result in several urogenital conditions including aerobic vaginitis [2]. The vaginal normal flora, especially lactobacilli, prevents other pathogenic organisms from colonizing the vagina and thereby helps in prevention of infection of vagina [3]. Disrupted vaginal microbiota forms a suitable basis for the development of complicated infections such as vulvovaginal candidiasis, trichomoniasis, and bacterial vaginosis. The untreated recurrent vaginal infections may lead to pre-term delivery, infertility, and other complications in the reproductive age group females [4]. Vaginitis may be caused by bacterial, fungal, or parasitic causative agents [5]. *Staphylococcus* and *Streptococcus* species may sometimes colonize the vagina and further lead to infections [6]. Aerobic vaginitis differs from bacterial vaginosis. It differs from the former in clinical features, laboratory diagnosis, and treatment [7]. Aerobic vaginitis is caused by *Staphylococcus aureus*, *Streptococcus agalactiae,* and *Enterobacteriaceae* [8]. The alteration of vaginal ecosystem contributes to the growth of pathogens which causes vaginal infections like bacterial vaginosis, sexually transmitted infections, and vulvovaginal candidiasis among others. Predisposing factors such as menstruation, pregnancy, sexual practice, uncontrolled usage of antibiotics, and vaginal douching can alter the microbial population and lead to infections. The most common bacterial causes of vaginal infections are *Gardnerella vaginalis*, *Treponema pallidum*, *Neisseria gonorrhoae*, and *Chlamydia trachomatis*, among others [3]. The diagnosis of vaginal infections is made by different methods like vaginal secretion wet mount examination, determination of the vaginal pH, whiff test, vaginal discharge culture, nucleic acid test [9]. In a study done among 657 patients with vaginal symptoms, there were cases of aerobic vaginitis, bacterial vaginosis, vulvovaginal candidiasis, trichomoniasis, and mixed infections. *Enterococcus faecalis*, *Streptococcus viridans*, *Escherichia coli*, and *Staphylococcus epidermidis* were frequently isolated [10]. 

The genus *Enterococcus* consists of Gram-positive, facultative anaerobic organisms that are ovoid in shape, arranged in short chains or in pairs. They were earlier classified as group D streptococcus [11]. *Enterococcus* is developing resistance against the most commonly used anti-enterococcal antibiotics like ampicillin and high-level aminoglycosides, besides being inherently resistant to many others like cephalosporins and clindamycin. This makes the treatment of these infections a real challenge for clinicians [12]. *Enterococcus* species is known for both intrinsic and acquired resistance to many antimicrobials. The most common mechanism for intrinsic resistance is due to the presence of many resistance genes against the various antimicrobials. The acquired resistance of enterococci is due to DNA mutation or acquiring of other new genes through different methods of gene transfer. This leads to development of resistance against many antibiotics like vancomycin, tetracycline, macrolides, fluoroquinolones, and others [13]. Multidrug resistant isolates are those isolates which are resistant to three or more different classes of antimicrobial agents [14]. In the last 50 years, there has been a rise in the multidrug-resistant (MDR) bacteria from both clinical and environmental specimens. These multidrug resistant organisms are also known as superbugs. The most dreaded multidrug resistant organisms are Gram negative bacilli like *Klebsiella pneumoniae*, *Acinetobacter baumannii*, *Pseudomonas aeruginosa*, and *Enterobacter* spp. On the other hand, among Gram positive bacteria, *Staphylococcus aureus* and *Enterococcus faecium* are pathogens in which multidrug resistance has also been reported. [15]. The bacteria have developed various mechanisms of resistance to the different antimicrobials. Among these mechanisms, the horizontal gene transfer of the resistance genes is an important one. Some bacteria also produce biofilms. These biofilms remain adherent to the surface and help the bacteria to evade the attack of the different antimicrobials [16]. Biofilm production is extremely important in recurrent bacterial infection. It protects the bacteria from antibiotics [17].

*Enterococcus* is a commensal bacterium of the gastrointestinal tract, but it can also become an opportunistic pathogen. It may colonize the female genital tract and vaginal colonization increases following antibiotic treatment or in patients with aerobic vaginitis. *E. faecalis* is associated with a wide spectrum of infections, particularly in immunocompromised states and when there is change in the host microbiota. There is increasing evidence which links enterococci with bacterial vaginosis and aerobic vaginitis [18].

There are very few studies regarding the association of vaginitis with *Enterococcus* isolates. This study was done to look for the biofilm production in *Enterococcus* isolates from cases of vaginitis. The antimicrobial susceptibility pattern along with the biofilm production was also looked for in these cases.

## 2. Materials and Methods

A prospective study was done in Medical College, Kolkata for a period of 2 years (from January 2018 to December 2019). The study was done after obtaining ethics clearance from the Institutional Ethics Committee. The adult female patients suspected of having vaginitis were considered for the study. Patients with genital ulcer were excluded from the study. An informed consent was obtained from the patients and clinical data were collected in a proforma.

### 2.1. Study Population and Preliminary Tests

Vaginal discharge was collected with sterile cotton swab and culture was done using routine bacteriological methods. The samples with growth of *Enterococcus* species were included in the study. Identification of enterococci was done preliminary by Gram stain, non-fastidious growth, and conventional biochemical tests like catalase test, growth on 6.5% NaCl, MacConkey agar, bile esculin agar, and arginine hydrolysis for genus identification and fermentation of mannitol, arabinose, sorbitol, and growth on tellurite agar and automated identification by VITEK 2 Compact (BioMerieux Inc., Marcy-l’Étoile, France) for species identification.

Urine samples were also collected from these selected patients to look for the presence of similar *Enterococcus* organisms. The urine samples were processed using standard microbiological techniques and identification and antimicrobial susceptibility of the organisms obtained with significant and probably significant bacteriuria were done.

### 2.2. Antimicrobial Susceptibility Test

These isolates were further tested for antimicrobial susceptibility to different antimicrobial agents like ampicillin (10 µg), tetracycline (30 µg), ciprofloxacin (5 µg), levofloxacin (5 µg), vancomycin (30 µg), teicoplanin (30 µg), linezolid (30 µg), and erythromycin by Kirby Bauer disc diffusion method using standard microbiological techniques on Mueller Hinton agar plates. Minimum inhibitory concentration (MIC) was tested by VITEK 2 Compact (BioMerieux Inc., France) for penicillin, tetracycline, ciprofloxacin, levofloxacin, vancomycin, teicoplanin, linezolid, and erythromycin. All interpretation of susceptibility pattern was done according to the Clinical and Laboratory Standards Institute (CLSI) version 2017 guidelines. The MIC for resistance of the antimicrobial agents were penicillin (≥16 µg/mL), tetracycline (≥16 µg/mL), ciprofloxacin (≥4 µg/mL), levofloxacin (≥8 µg/mL), vancomycin (≥32 µg/mL), teicoplanin (≥32 µg/mL), linezolid (≥8 µg/mL), and erythromycin (≥8 µg/mL). Susceptibility to high level gentamicin (120 µg) was done by Kirby Bauer disc diffusion method and interpretation was done by using EUCAST guidelines version 2016. The quality control for antimicrobial susceptibility testing was done with *Staphylococcus aureus* ATCC 25923 for disc diffusion and *Enterococcus faecalis* ATCC 29212 for dilution method.

### 2.3. Detection of Biofilm 

All the clinical isolates included in the study were tested for biofilm production by the procedure used by Kafil and Mobarez in 2013 and Triveda and Gomathi in 2016. The strains of *Enterococcus* were stored at −20 °C. These strains were freshly subcultured on blood agar. Then the isolates were inoculated in 1 mL of Brain Heart Infusion (BHI) broth with 1% glucose. The inoculated BHI broth was incubated at 37 °C for 24 h. After 24 h of incubation, 20 μL of this growth containing BHI broth was added to 180 μL of fresh BHI broth, and the turbidity was checked corresponding to a turbidity of 0.5 McFarland standard. The control strain used was *E. faecalis* ATCC 29212. For both the control and test strains, 200 μL of each of the isolate suspension were placed into flat bottom microtiter plates. All isolates were inoculated in duplicates and incubated at 37 °C in 5% CO_2_ for 24 h. After 24 h of incubation, the contents of the plates were discarded. Each well was thoroughly washed three to five times with phosphate buffered saline, tapped, and dried. Then 150 µL of methanol was added to each of the wells and kept for 20 min. This was done for fixation of the biofilm. After that, the fixed biofilm was dried in air by keeping it for about 30 min in an inverted position. Then the biofilm was stained with 0.1% crystal violet for 15 min. The excess of stain was discarded. The plates were thoroughly washed with distilled water. Finally, 150 μL of 33% acetic acid was added to each well and kept for 30 min without shaking. The optical density (OD) was measured at 570 nm. Based on the OD values, the isolates were categorized as strong biofilm producers (OD 570 > 2), moderate biofilm producer (OD 570 > 1 but <2), weak biofilm producer (OD 570 > 0.5 but <1), and non-biofilm producers (OD 570 ≤ 0.5) [19].

### 2.4. Data Analysis

The clinical and test data were entered in the Microsoft Excel spreadsheet (Microsoft Office, Washington, WA, USA). The geometric mean (GM) and the standard deviation (SD) were calculated using excel spreadsheet for the numerical variables. All statistical analysis was done using STATA version 20. The data were summarized using mean along with standard deviation for continuous variables, and frequency along with percentages for categorical variables. Chi square test was used to check the categorical variables association and *p* value < 0.05 was taken as significant.

## 3. Results

During the period of 2 years (from January 2018 to December 2019), there were 39 isolates of *Enterococcus* obtained from the cases of vaginitis. The clinical profiles of the patients are shown in Table 1. Most of the patients were in the reproductive age group. All patients had vaginal discharge; 22 patients had itching. 

Among these 27 (69.23%) were *Enterococcus faecalis* and 12 (30.77%) *Enterococcus faecium*. All isolates were highly susceptible to vancomycin, teicoplanin, and linezolid. Only one isolate (2.56%) was resistant to vancomycin and teicoplanin and it was Van A type. Table 2 shows the susceptibility of the *Enterococcus* isolates to the different antimicrobial agents. 

Only three patients with vaginal discharge had growth of the similar *Enterococcus* species from urine sample. The colony count for all three urine samples was 10^4^ CFU/mL. The antimicrobial susceptibility for these isolates were exactly like that of the one obtained from vaginal discharge. Among the rest of the urine samples, 34 samples showed no growth of any organism and 2 samples showed growth of *Escherichia coli*.

Biofilm was detected in eight (20.51%) isolates of which five were strong biofilm producer and three moderate biofilm producers. There were 31 non biofilm producer isolates. Among the 39 isolates, 27 (69.23%) were multidrug resistant, that is, resistant to three different classes of antibiotics. All eight isolates of biofilm-producing *Enterococcus* were multidrug resistant. Among these eight biofilm-producing enterococci isolates, six were *Enterococcus faecium* and two were *Enterococcus faecalis*. There was a significant association of biofilm production in *Enterococcus faecium* (*p* = 0.0056). There was also a strong association of biofilm production in multidrug resistant isolates as compared to the more susceptible isolates (*p* = 0.0417). There was only one isolate of vancomycin resistant *Enterococcus* which was resistant to both vancomycin and teicoplanin.

## 4. Discussion

Bacterial vaginosis is related to a change in vaginal tract ecology, which includes a decrease in the concentration and/or prevalence of facultative lactobacilli. In a study by Kelly et al., it was found that one specific strain of *Enterococcus faecium* may cause decline in the population of lactobacilli, thus indirectly favoring the development of bacterial vaginosis [20]. In this study, 39 isolates of *Enterococcus* were isolated from vaginal discharge. Most of the women were in the reproductive age group (89.7%). Only five patients were pregnant. The common organisms present in the vagina include *Lactobacillus*, the predominant commensal, and others like *Corynebacterium*, *Mobiluncus*. anaerobic bacteria; and, rarely, *Streptococcus* and *Staphylococcus*. These organisms play a major role in maintaining the low pH of the vagina and prevent the colonization of vagina by other pathogenic bacteria [21]. In this study the *Enterococcus* isolates obtained from the vaginal discharge were included.

In a study done by Chakroborty et al. in Kolkata in 2011, there was a prevalence of 7.3% *Enterococcus* isolates from all clinical samples [22]. In a study done in Assam, speciation of 93 *Enterococcus* species by Vitek 2 automated system was like that by conventional biochemical tests. *E. faecalis* was the commonest species (81.72%) isolated, followed by *E. faecium* (12.9%), *E. raffinosus* (3.23%, *n* = 3), *E. avium* (1.08%, *n* = 1), and *E. gallinarum* (1.08%, *n* = 1) [23]. In this study, among these 39 isolates, 27 (69.23%) were *Enterococcus faecalis* and 12 (30.77%) were *Enterococcus faecium*. Similar results were seen in another study in Uttar Pradesh which found that out of 100 *Enterococcus* strains, 47 were *E. faecalis*, 51 were *E. faecium*, two were *E. gallinarum*, and one was *E. casseliflavus* [24].

*Enterococcus* has developed glycopeptide resistance. Some *Enterococcus* like *Enterococcus gallinarum* and *Enterococcus casseliflavus/flavescens* has intrinsic, but low-level vancomycin resistance. For such resistance there is presence of the VanC-1 ligase in case of *E. gallinarum*, and VanC-2/3 ligase in case of *E. casseliflavus/flavescens*. The peptidoglycan precursors, especially those with D-alanyl-D-serine, require VanC enzyme for their synthesis. Organisms which have VanC are resistant to vancomycin but are susceptible to teicoplanin. This naturally occurring vancomycin resistance is chromosomally encoded and cannot be transferred from one organism to another [25]. In this study there was only one isolate of *E. faecium* resistant to vancomycin and teicoplanin. 

There are studies showing the relationship between urinary tract infection (UTI) and the gut flora. Many times, it has occurred that the UTI causing pathogen is same species with similar antimicrobial resistance pattern as that of the gut flora. Most of these studies are done from UTI patients and obtain similar organisms from gut, vaginal, or urethral flora. This occurs as the gut flora colonizes the urethra and then the organism ascends in a retrograde manner from the urethra, thereby causing UTI [26]. In this study, we found similar *Enterococcus* species of organism from the vaginal discharge and urine isolate in three patients. The most common Gram-positive organisms causing UTI are *Staphylococcus aureus*, *Staphylococcus saprophyticus*, *Enterococcus* species, Group B Streptococcus, or *Streptococcus agalactiae*. The Gram-positive organisms may even be the causative agent of UTI as a monomicrobial or a polymicrobial agent [27]. 

In this study the prevalence of multidrug resistant *Enterococcus* was 27 (69.23%). This is similar to the finding of Bhatt et al. where the prevalence of multidrug resistance among *Enterococcus* was found to be 63% among 200 clinical isolates [28]. In a study done by Praharaj and colleagues, out of 367 isolates of *Enterococcus*, 32 (8.7%) were found to be resistant to vancomycin. None of the *Enterococcus* isolates were resistant to linezolid [29]. However, in this study there was only one isolate of vancomycin resistant *Enterococcus*. This finding is less in comparison to the findings in Mangalore, where out of 150 total isolates, 13 (8.6%) isolates showed vancomycin resistance, of which 11 (7.3%) had an MIC > 8 μg/mL [30]. Though the first report of linezolid resistant *Enterococcus* isolate was from Kolkata [31], we did not find any linezolid resistant strain. In a study done to look for the antimicrobial resistance pattern of the *Enterococcus* species from vaginal flora, minimum inhibitory concentration for different antibiotics was performed and all *Enterococcus* isolates were susceptible to gentamicin, streptomycin, ampicillin, penicillin, vancomycin, linezolid, erythromycin, and chloramphenicol. Among the *E. faecalis* isolates, there was less susceptibility seen in case of tetracycline, clindamycin, and quinupristin-dalfopristin [32]. 

*Enterococcus faecalis* is known to produce biofilms which makes it resistant to the different antimicrobial agents [33]. *Pseudomonas aeruginosa* and *Enterococcus faecalis* isolated from chronic infections are two bacteria which produce biofilms as a virulence factor for them [34]. In this study, most of the biofilm producing isolates were *Enterococcus faecium.* The biofilm producing strains of *Enterococcus* were more resistant than the non-biofilm producing strains. This is similar to the findings of Anna Sienko where antimicrobial resistance was significantly related to biofilm production [35]. Another study also showed that antimicrobial resistance was more common in the biofilm producing *Enterococcus* isolates [36]. In the current study multidrug resistance had a significant association with the biofilm production.

## 5. Conclusions

Biofilm production is an important virulence factor among the multidrug resistant enterococcus isolates. The clinicians should be on constant vigil to look for multidrug resistant isolates as it is extremely difficult to eradicate these isolates. Moreover, larger prospective studies need to be conducted to look for the association of biofilm production with other risk factors.

## Figures and Tables

**Table 1:** Clinical profile of the patients.

Characteristics	Present (%)
Age (18–50 years)	35 (89.7%)
Pregnancy	5 (12.8%)
Vaginal discharge	39 (100%)
Fever	4 (10.25%)
Itching	22 (56.4%)

**Table 2:** Antimicrobial susceptibility of *Enterococcus* spp. (*n* = 39).

Antimicrobial Agent	*Enterococcus* spp. (*n* = 39)	MIC Range μg/mL	MIC_90_ μg/mL	MIC_50_ μg/mL
Ampicillin/Penicillin	9 (23.08%)	≤2–≥32	32	32
Ciprofloxacin	10 (25.64%)	≤0.5–≥8	8	8
Levofloxacin	12 (30.77%)	≤0.5–≥8	8	8
High level gentamicin	24 (61.54%)	-	-	-
Erythromycin	30 (76.92%)	≤16–256	128	16
Vancomycin	38 (97.43%)	≤0.5–≥32	1	0.5
Teicoplanin	38 (97.43%)	≤0.5–≥32	1	0.5
Linezolid	39 (100%)	≤0.5–4	1	0.5
Tetracycline	8 (20.51%)	≤0.5–≥16	16	16

## References

[1] Hainer B.L., Gibson M.V. (2011). Vaginitis: Diagnosis and Treatment. Am. Fam. Physician.

[2] Kaambo E., Africa C., Chambuso R., Passmore J.-A.S. (2018). Vaginal Microbiomes Associated with Aerobic Vaginitis and Bacterial Vaginosis. Front. Public Health.

[3] Chee W.J.Y., Chew S.Y., Than L.T.L. (2020). Vaginal microbiota and the potential of Lactobacillus derivatives in maintaining vaginal health. Microb. Cell Fact..

[4] Kalia N., Singh J., Kaur M. (2020). Microbiota in vaginal health and pathogenesis of recurrent vulvovaginal infections: A critical review. Ann. Clin. Microbiol. Antimicrob..

[5] Cullins V.A., Dominguez L., Guberski T., Secor R.M., Wysocki S.J. (1999). Treating vaginitis. Nurse Pract..

[6] Xu J., Bian G., Zheng M., Lu G., Chan W., Li W., Yang K., Chen Z., Du Y. (2020). Fertility factors affect the vaginal microbiome in women of reproductive age. Am. J. Reprod. Immunol..

[7] Sonthalia S., Aggarwal P., Das S., Sharma P., Sharma R., Singh S. (2020). Aerobic vaginitis—An underdiagnosed cause of vaginal discharge—Narrative review. Int. J. STD AIDS.

[8] Gajdács M., Urbán E. (2019). Epidemiology and resistance trends of Staphylococcus aureus isolated from vaginal samples: A 10-year retrospective study in Hungary. Acta Dermatovenerol. Alp. Pannonica Adriat..

[9] Quan M. (2010). Vaginitis: Diagnosis and management. Postgrad. Med..

[10] Fan A., Yue Y., Geng N., Zhang H., Wang Y., Xue F. (2013). Aerobic vaginitis and mixed infections: Comparison of clinical and laboratory findings. Arch. Gynecol. Obstet..

[11] Murray B.E. (1990). The life and times of the Enterococcus. Clin. Microbiol. Rev..

[12] Gupta V., Singla N., Behl P., Sahoo T., Chander J. (2015). Antimicrobial susceptibility pattern of vancomycin resistant enterococci to newer antimicrobial agents. Indian J. Med. Res..

[13] Esmail M.A.M., Abdulghany H.M., Khairy R.M. (2019). Prevalence of Multidrug-Resistant Enterococcus faecalis in Hospital-Acquired Surgical Wound Infections and Bacteremia: Concomitant Analysis of Antimicrobial Resistance Genes. Infect. Dis..

[14] Falagas M.E., Karageorgopoulos D.E. (2008). Pandrug Resistance (PDR), Extensive Drug Resistance (XDR), and Multidrug Resistance (MDR) among Gram-Negative Bacilli: Need for International Harmonization in Terminology. Clin. Infect. Dis..

[15] Uruén C., Chopo-Escuin G., Tommassen J., Mainar-Jaime R.C., Arenas J. (2020). Biofilms as Promoters of Bacterial Antibiotic Resistance and Tolerance. Antibiotics.

[16] Lebeaux D., Ghigo J.-M., Beloin C. (2014). Biofilm-related infections: Bridging the gap between clinical management and fundamental aspects of recalcitrance toward antibiotics. Microbiol. Mol. Biol. Rev..

[17] Jung H.-S., Ehlers M.M., Lombaard H., Redelinghuys M.J., Kock M.M. (2017). Etiology of bacterial vaginosis and polymicrobial biofilm formation. Crit. Rev. Microbiol..

[18] Alhajjar N., Chatterjee A., Spencer B.L., Burcham L.R., Willett J.L.E., Dunny G.M., Duerkop B.A., Doran K.S. (2020). Genome-Wide Mutagenesis Identifies Factors Involved in Enterococcus faecalis Vaginal Adherence and Persistence. Infect. Immun..

[19] Shridhar S., Dhanashree B. Antibiotic Susceptibility Pattern and Biofilm Formation in Clinical Isolates of *Enterococcus* spp.. https://www.hindawi.com/journals/ipid/2019/7854968/.

[20] Kelly M.C., Mequio M.J., Pybus V. (2003). Inhibition of Vaginal Lactobacilli by a Bacteriocin-Like Inhibitor Produced by Enterococcus faecium 62-6: Potential Significance for Bacterial Vaginosis. Infect. Dis. Obstet. Gynecol..

[21] Gupta P., Singh M.P., Goyal K. (2020). Diversity of Vaginal Microbiome in Pregnancy: Deciphering the Obscurity. Front. Public Health.

[22] Chakraborty A., Pal N., Sarkar S., Gupta M. (2015). Antibiotic resistance pattern of Enterococci isolates from nosocomial infections in a tertiary care hospital in Eastern India. J. Nat. Sci. Biol. Med..

[23] Barman J., Nath R., Saikia L. (2016). Drug resistance in Enterococcus species in a tertiary level hospital in Assam, India. Indian J. Med. Res..

[24] Jaiswal S., Singh A., Verma R.K., Singh D.P., Kumari S. (2017). Characterization, speciation and antimicrobial resistance pattern of Enterococcus species isolated from clinical specimens at a rural tertiary care hospital. Int. J. Res. Med. Sci..

[25] Reid K.C., Cockerill F.R., Patel R. (2001). Clinical and Epidemiological Features of Enterococcus casseliflavus/flavescens and Enterococcus gallinarum Bacteremia: A Report of 20 Cases. Clin. Infect. Dis..

[26] Meštrović T., Matijašić M., Perić M., Čipčić Paljetak H., Barešić A., Verbanac D. (2020). The Role of Gut, Vaginal, and Urinary Microbiome in Urinary Tract Infections: From Bench to Bedside. Diagnostics.

[27] Kline K.A., Lewis A.L. (2016). Gram-Positive Uropathogens, Polymicrobial Urinary Tract Infection, and the Emerging Microbiota of the Urinary Tract. Microbiol. Spectr..

[28] Bhatt P., Patel A., Sahni A.K., Praharaj A.K., Grover N., Chaudhari C.N., Das N.K., Kulkarni M. (2015). Emergence of multidrug resistant enterococci at a tertiary care centre. Med. J. Armed Forces India.

[29] Praharaj I., Sujatha S., Parija S.C. (2013). Phenotypic & genotypic characterization of vancomycin resistant Enterococcus isolates from clinical specimens. Indian J. Med. Res..

[30] Fernandes S.C., Dhanashree B. (2013). Drug resistance & virulence determinants in clinical isolates of Enterococcus species. Indian J. Med. Res..

[31] Kumar S., Bandyoapdhyay M., Chatterjee M., Mukhopadhyay P., Poddar S., Banerjee P. (2014). The first linezolid-resistant Enterococcus faecium in India: High level resistance in a patient with no previous antibiotic exposure. Avicenna J. Med..

[32] Sirichoat A., Flórez A.B., Vázquez L., Buppasiri P., Panya M., Lulitanond V., Mayo B. (2020). Antibiotic Resistance-Susceptibility Profiles of *Enterococcus faecalis* and *Streptococcus* spp. From the Human Vagina, and Genome Analysis of the Genetic Basis of Intrinsic and Acquired Resistances. Front. Microbiol..

[33] Kumar L., Cox C.R., Sarkar S.K. (2019). Matrix metalloprotease-1 inhibits and disrupts Enterococcus faecalis biofilms. PLoS ONE.

[34] Lee K., Lee K.-M., Kim D., Yoon S.S. (2017). Molecular Determinants of the Thickened Matrix in a Dual-Species Pseudomonas aeruginosa and Enterococcus faecalis Biofilm. Appl. Environ. Microbiol..

[35] Sieńko A., Wieczorek P., Majewski P., Ojdana D., Wieczorek A., Olszańska D., Tryniszewska E. (2015). Comparison of antibiotic resistance and virulence between biofilm-producing and non-producing clinical isolates of Enterococcus faecium. Acta Biochim. Pol..

[36] Woźniak-Biel A., Bugla-Płoskońska G., Burdzy J., Korzekwa K., Ploch S., Wieliczko A. (2019). Antimicrobial Resistance and Biofilm Formation in Enterococcus spp. Isolated from Humans and Turkeys in Poland. Microb. Drug Resist..

